# Safety of oil transportation through road- issues & recommendations 

**Published:** 2019-08

**Authors:** Ajmal Khan Khoso

**Affiliations:** ^*a*^National Highways & Motorway Police, Pakistan.

## Abstract

Transportation of oil through road has history of safety issues in developing nations, which in ‎some instances resulted in heavy toll and economic loss. Recent such incident took place in ‎Pakistan in June, 2017 which resulted in death of more than 200 people. There is need to ‎analyze oil transportation mechanism through roads in developing countries to address the issue ‎of safety in order to control such deteriorations which result in heavy losses in terms of human ‎lives and property damages. The guidelines will be helpful in preventing road crashes of oil ‎tankers and if a case of an accident will be helpful in minimizing the loss. These guidelines ‎have been formulated on the basis of Hiddon’s Matrix and recommendations of Word Report of ‎Injury Prevention 2004. Moreover; evidence based best practices on the subject issue have also ‎been searched and incorporated in the report. Multifaceted recommendations have been ‎formulated based upon various factors and elements such as on the basis of transportation ‎planning, the burden of oil transportation on road need to be reduced and segregated to railways ‎and pipeline on safety and economy basis; enforcement mechanisms need to be strengthened by ‎provision of special authorities for regulations of transportation companies, vehicles safety ‎design, fitness and driving licensing; special trainings for first responders and drivers for ‎handling hazardous materials crises; provision of enforcement of driver’s working hours and ‎establishment of rest areas to address issue of drowsiness and fatigue ; and lastly establishment ‎of firefighting center and burn centers at divisional units for handling post-crash issues such a s ‎fire and burn injuries. ‎

The report addresses wide range of issues pertaining to road transportation of oil in developing ‎countries and recommendations can be helpful in prevention of related road crashes, post-crash ‎management and addressing associated consequences. Implementation of recommendations will ‎improve safety of oil carriers and will be helpful in reducing related road crashes.‎

**Keywords:**

Oil Transportation safety

